# Treatment of unexplained facial numbness with stellate ganglion blockade: A case report

**DOI:** 10.1002/ccr3.6871

**Published:** 2023-01-19

**Authors:** Uno Imaizumi, Satoshi Beppu, Takuro Sanuki

**Affiliations:** ^1^ Department of Dental Anesthesiology Kanagawa Dental University Yokosuka Kanagawa Japan

**Keywords:** dysesthesia, hypesthesia, numbness, stellate ganglion blockade

## Abstract

To date, there is no established treatment for facial numbness or dysesthesia of unspecified causes. Herein, we report a case of unexplained facial numbness and confirmed hypesthesia that achieved clinical response to stellate ganglion blockade (SGB). SGB might be an effective treatment of psychological stress‐related facial numbness of unknown origin.

## Abstract 2

To date, there is no established treatment for facial numbness or dysesthesia of unknown origin. A 50‐year‐old man with no history of psychosomatic consultation and a tendency toward anxiety on the Hospital Anxiety and Depression Scale presented with facial numbness. He was successfully treated using a stellate ganglion block (SGB).

## 1 INTRODUCTION

Numbness and sensory disturbances of the face due to peripheral neuropathy caused by tumors, inflammation, or surgical nerve damage can be easily diagnosed.^1^ An approach to managing primary disease is the chosen treatment strategy. Stellate ganglion blockade (SGB) is often the treatment of choice for paresthesia due to nerve damage. However, there is no established treatment of numbness or dysesthesia of undetermined origin. Herein, we report a case of clinical improvement after SGB sessions in a patient with unexplained facial numbness and confirmed hypesthesia.

## 2 CASE REPORT

### 2.1 Patient information

A 50‐year‐old man with an unremarkable medical history presented with numbness in the skin of the upper lip around the right anterior maxillary teeth and from the area around the eye to the cheekbone. The numbness had persisted for a month. The patient was referred to our department for an evaluation of these unexplained symptoms.

### 2.2 Clinical findings

**FIGURE 1:**
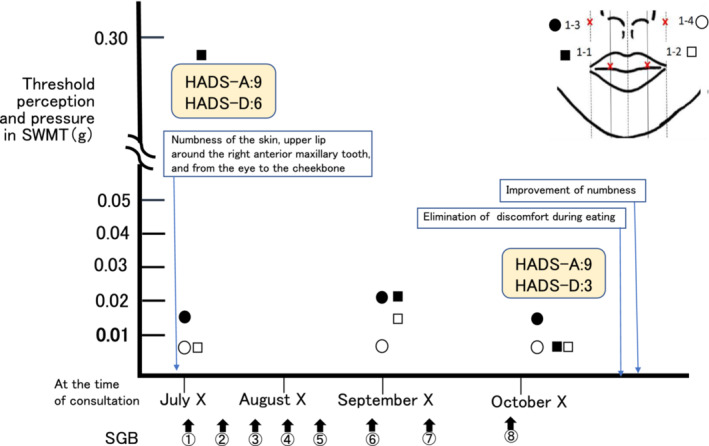
Timeline

At the first visit to our department, we performed the Semmes–Weinstein monofilament test (SWMT), a non‐invasive method of measuring tactile perception thresholds using monofilaments. This test revealed a threshold of 0.28 g on the right upper lip (Figure 1, 1‐1) and 0.014 g on the right lateral nasal wing skin (Figure 1, 1‐3). The thresholds of the healthy contralateral areas (Figure 1, 1‐2 and 1‐4) were both 0.008 g. The scores of the Japanese version of the Hospital Anxiety and Depression Scale (HADS)^2^ that was used to assess anxiety (HADS‐A) and depression (HADS‐D) were 9 and 6, respectively. No complaints of pain were reported.

### 2.3 Timeline

The timeline is shown in Figure 1.

### 2.4 Diagnostic assessment

The patient was diagnosed with trigeminal neuropathy of the second branch of the right trigeminal nerve and right‐sided maxillary hypesthesia. Therefore, a right‐sided SGB was performed.

### 2.5 Therapeutic intervention

The treatment course included a right‐sided SGB using 5 ml of mepivacaine (1%) for this trigeminal neuropathy. SGB was performed once a week, and the three main signs of Horner's syndrome (ptosis, pupil contraction, and ocular depression) were observed. The SGB was administered after identifying possible mental stressors using the HADS. The changes following SGB administration were also observed, including a reduction in the heart rate and deep sleep, which confirmed the relaxation of the sympathetic nervous system. In addition, during the initial interview and at each visit, the patient's concerns about changes in the work environment were listened to.

### 2.6 Follow‐up and outcomes

Two months after the first visit, the threshold of the skin of the right upper lip and the right lateral nasal wing was 0.02 g according to the SWMT. After six sessions of SGB, the healthy contralateral areas showed an improvement in the upper lip and lateral nasal wing of 0.014 g and 0.008 g, respectively. After eight sessions of SGB, the numbness had improved as a subjective symptom. Since discomfort during eating disappeared, the treatment was terminated. HADS‐A remained unchanged at 9 points. HADS‐D decreased to 3 points.

## 3 DISCUSSION

By temporarily blocking the sympathetic nervous system, SGB causes dilation of the internal carotid and vertebral arteries, which run through the brain and spinal cord. The resulting increase in blood flow not only improves pain associated with local circulatory disturbances (ischemia) but also has a positive effect on the hypothalamic function, thereby promoting neural recovery. SGB is used for the treatment of migraine, upper limb pain, and atypical facial pain.^3^ Since SGB is used for the treatment of many systemic diseases, including those of the immune and endocrine systems, it has a wider range of indications in Japan than in Europe or the United States.^4^

Trigeminal neuropathy is a general term for a condition in which the trigeminal nerve becomes dysfunctional due to trauma, tumors, infectious, or demyelinating diseases. Trigeminal neuropathy results in sensory abnormalities, with or without pain.^5^ In the present case, there was no pain at the time of the initial examination. However, sensory abnormalities were observed, leading to a diagnosis of trigeminal neuropathy.

Although there is no established treatment for unexplained paresthesia and numbness, SGB was effective, in this case, in which SWMT showed improvement in the threshold. There are a few reports on the treatment of unexplained paresthesia. The mechanism of the therapeutic effect of SGB in this case may be similar to that in complex regional pain syndrome and post‐traumatic stress disorder. Nerve growth factor (NGF) increases in response to acute or chronic stress. It promotes the growth of the sympathetic nerve endings and increases noradrenaline levels in the brain. SGB exerts a therapeutic effect by reducing the levels of NGF and noradrenaline.^3^, ^6^ Acupuncture treatment could alter the secretion patterns of noradrenaline, gamma‐aminobutyric acid, and serotonin.^6^ Furthermore, an increased sympathetic tone could reduce the function and sensitivity of pinealocytes, attenuating melatonin rhythms and causing various diseases. However, the mechanism of action of SGB comprises sympathetic inhibition within the innervated area, causing vasodilation. This not only increases blood flow but also triggers the restoration of melatonin secretion by the pineal gland, normalizing the physiological melatonin rhythms.^4^ Regarding the treatment of stress‐related illnesses, mindfulness may be effective in reducing stress by decreasing sympathetic activity and increasing parasympathetic activity.^7^ SGB induces a parasympathetic‐dominant state, which is characterized by peripheral blood vessel dilation, increases in blood flow and respiratory rate, and a decrease in heart rate. In the present case, deep sleep and reduced heart rate were also observed after each SGB session, indicating a relaxing effect due to sympathetic suppression.

In this case, although there was no apparent etiology for numbness, there was a change in the work environment due to the COVID‐19 pandemic restrictions. The onset of numbness occurred in a more stressful situation than before. The post‐SGB HADS assessment showed improvement in the depression rating scale. In addition, numbness could be a symptom of somatization disorder or depression.^8^ Therefore, it is possible that numbness, in this case, may have developed due to psychological factors. As this was a report of a single case, further research on patients with other possible psychological factors and those without any psychological factors is necessary to confirm the utility of SGB in treating facial numbness of unknown etiology.

## 4 CONCLUSION

A decrease in heart rate and deep sleep after SGB showed a relaxing effect due to sympathetic suppression and an improvement in the depression rating scale. These results suggest that SGB might be effective in the treatment of facial numbness related to mental stress.

## AUTHOR CONTRIBUTIONS

**Uno Imaizumi:** Conceptualization; data curation; formal analysis; investigation; writing – original draft. **Satoshi Beppu:** Validation; writing – review and editing. **Takuro Sanuki:** Validation; writing – review and editing.

## FUNDING INFORMATION

None.

## CONFLICT OF INTEREST

The authors declare no conflicts of interest associated with this manuscript.

## ETHICAL APPROVAL

This case report was approved by the Institutional Ethics Committee (No. 868) of the Kanagawa Dental University in accordance with the Declaration of Helsinki. This report is presented in accordance with the CARE guidelines established by the EQUATOR network.

## CONSENT

Written informed consent was obtained from the patient to publish this report in accordance with the journal's patient consent policy.

## Data Availability

Data sharing is not applicable to this article, as no datasets were generated or analyzed during the current study.
